# Enhancing the Dyeability of Polyimide Fibers with the Assistance of Swelling Agents

**DOI:** 10.3390/ma12030347

**Published:** 2019-01-22

**Authors:** Dongyan Shao, Changhai Xu, Hongbo Wang, Jinmei Du

**Affiliations:** Key Laboratory of Eco-textiles, Ministry of Education, College of Textiles and Clothing, Jiangnan University, Jiangsu 214122, China; dongyan-shao@foxmail.com (D.S.); changhai_xu@jiangnan.edu.cn (C.X.)

**Keywords:** Polyimide fiber, thermal stability, swelling agent, dyeability

## Abstract

Polyimide (PI) fibers have outstanding thermal stability and mechanical properties, but are difficult to dye with disperse and basic dyes. In this work, it was proposed to use N-methylformanilide (MFA), phenoxyisopropanol (PIP), and acetophenone (AP) as swelling agents to enhance the dyeability of PI fibers. The PI fibers treated with swelling agents were characterized by thermal gravimetric analysis, scanning electronic microscopy, tensile testing, and crystalline analysis. It was found that the swelling agents penetrated into the PI fibers in amounts greater than 10% (pertaining to the weight of PI fibers). The swelling agents did not really swell the PI fibers, but broke the interaction forces between the PI macromolecules. With the assistance of swelling agent, the PI fibers could be dyed with disperse and basic dyes in strong color strengths. AP exhibited the best performance for enhancing the dyeability of PI fibers, followed by MFA and PIP. The dyed PI fibers were found to have good colorfastness to washing.

## 1. Introduction

Aromatic Polyimides (PIs) are a series of heterocyclic polymers synthesized by polycondensation of dianhydrides with diamines, and exhibit excellent thermal stability because of their cyclic and rigid molecular chain backbones [1,2,3]. So far PIs have been applied as high-performance thermally stable materials for films, membranes, coatings, and fibers [4,5,6,7,8,9]. As spinning technologies have advanced in enhancing the mechanical properties of PI fibers [10,11,12], PI fibers have been proposed for use in textiles such as thermal protective clothing for firefighters [13,14]. However, PI fibers are difficult to dye with common dyestuffs due to the fact that they contain few functional groups for dyeing, and also the highly compact arrangement of the PI macromolecular backbones limits their interactions with dye molecules. A facile approach to coloration of PI fibers is dope-dyeing which is carried out by mixing pigments or dyestuffs with PI before spinning. However, the dope-dyeing method has drawbacks of reduction in stability of the spinning solution and spinneret clogging. It has been recently reported that the dyeability of PI fibers for disperse dyes can be improved by pretreatment with alkali [15,16], but PI fibers undergo apparent strength loss due to alkali hydrolysis. The swelling agents are so called because they can expand the amorphous region in the fibers for easier penetration of dye molecules, so that the fiber can be dyed at a lower temperature. Coloration with swelling agents has been well studied and practiced on polyester, aramid, and other synthetic fibers [17,18,19,20]. Anilide, phenoxy, and phenone types of compounds are often used as swelling agents because these organic compounds are hydrophobic with strong polarity which provides them with intense interactions with fibers. In this work, it was proposed that PI fibers be dyed with disperse dyes and basic dyes by using *N*-methylformanilide (MFA), phenoxyisopropanol (PIP), and acetophenone (AP) as swelling agents. The properties of the dyed PI fibers were evaluated by color measurement, thermal analysis, crystalline analysis, and surface morphology.

## 2. Materials and Methods

### 2.1. Materials

**Figure 1:**
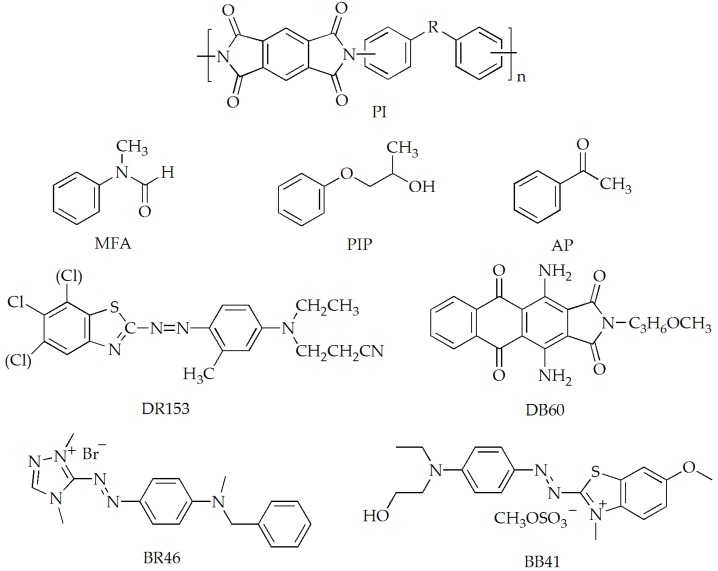
Chemical structures of Polyimide (PI) fibers, dyes and swelling agents.

PI fibers in double yarns (29.2 tex) were provided by Aoshen New Material Inc. (Jiangsu, China). C.I. Disperse Red 153 (DR 153), C.I. Disperse Blue 60 (DB 60), C.I. Basic Red 46 (BR 46) and C.I. Basic Blue 41 (BB 41) were used for dyeing of PI fibers, and provided by DyStar (Shanghai, China). MFA, PIP, and AP were used as swelling agents, and provided by Sigma-Aldrich (Shanghai, China). Figure 1 shows the chemical structures of PI fibers, dyes and swelling agents. Soaping agent, hydrosulphite (Na_2_S_2_O_4_) and sodium hydroxide (NaOH) were used as auxiliaries for soaping and reduction clearing of the dyed PI fibers. 

### 2.2. Dyeing of PI Fibers

Dyeing experiments were carried out on an Ahiba IR dyeing machine (Datacolor, Lawrenceville, NJ, USA). PI fibers were dyed at a liquor-to-material ratio of 20:1 with 5% of disperse and basic dyes (owf) by adding 50 g/L of swelling agents. The temperature of the dyebath was raised to 130 °C at a rate of 2 °C/min, and held for 60 min. When the dyeing was complete, the temperature was lowered to 85 °C, and the dyed PI fibers were taken out for washing. The PI fibers dyed with basic dyes were washed by soaping with 2 g/L of soaping agent at 85 °C for 20 min, and the PI fibers dyed with disperse dyes were washed by reduction cleaning with 2 g/L of hydrosulphite and 2 g/L of sodium hydroxide at 85 °C for 20 min. All the dyed PI fibers were rinsed thoroughly with fresh water and dried under ambient conditions.

### 2.3. Testing of PI Fibers

#### 2.3.1. Thermal Analysis

Thermal degradation behavior of PI fibers was tested on a TA Q500 thermal analysis machine (TA Instruments, New Castle, DE, USA) in a nitrogen atmosphere, and the heating rate was 20 °C/min with the temperature ranging from 50 °C to 900 °C.

#### 2.3.2. Surface Morphology

PI fibers were sputter-coated with gold and scanned for surface morphology with a magnification of 3000 through a SU1510 scanning electron microscope (Hitachi, Tokyo, Japan).

#### 2.3.3. Tensile Properties

Tensile strength and breaking elongation of PI fibers were measured in terms of the ISO 2062-2009 on the YG020B Single Yarn Tensile Tester (Futai Machinery Co., Ltd, Changzhou, China) which was set with a drawing speed of 250 mm/min and a test length of 250 mm. All samples were measured 20 times to give an average value.

#### 2.3.4. Crystalline Analysis

The crystallinity of PI fibers before and after treating with swelling agents was analyzed through an AXS D8 wide angle X-ray diffractometer (Bruker, Karlsruher, German) (WAXD) with a scan angel 2θ of 10° to 40° at a scanning speed of 5°/min.

#### 2.3.5. Color Strength

The dyed PI yarns were knitted into single jersey circular fabric (121 g/m^2^). The reflectance of the dyed PI fabric was measured on a Datacolor 650 Spectrophotometers (Datacolor, Lawrenceville, NJ, USA) under the CIE Standard Illuminant D65 and the CIE 1964 10° Standard Observer. The color strength(K/S) of the dyed fibers was calculated from the reflectance by the Kubleka–Munk equation as shown in Equation (1),

(1) $$K/S=\frac{{\left(1.0-R_{\lambda }\right)}^{2}}{2R_{\lambda }}$$

where K is the absorption coefficient, S is the scattering coefficient, and R*_λ_* is the reflectance at a specific wavelength (*λ*) ranging from 400 to 700 nm.

#### 2.3.6. Colorfastness to Washing of the Dyed PI Fibers

The colorfastness to washing of the dyed PI fibers was tested in terms of ISO 105-C10: 2006 (https://www.iso.org/standard/31775.html, accessed on 10 September 2017). The dyed yarns were stitched to a multifiber adjacent fabric containing wool, acrylic, polyester, polyamide, cotton, and acetate, and washed in a solution containing 5 g/L of a standard detergent (ECE) and 2 g/L of sodium carbonate at 60 °C for 30 min. Colorfastness rating was estimated by using the AATCC grey scale.

## 3. Results and Discussion

### 3.1. Effect of Swelling Agents on PI Fibers

It was assumed that the swelling agents play the role of enhancing the dyeability of PI fibers by penetrating into the PI fibers and expanding the pore apertures in the PI fibers. This assumption was confirmed by characterizing the physical and mechanical properties of PI fibers treated with swelling agents.

**Figure 2:**
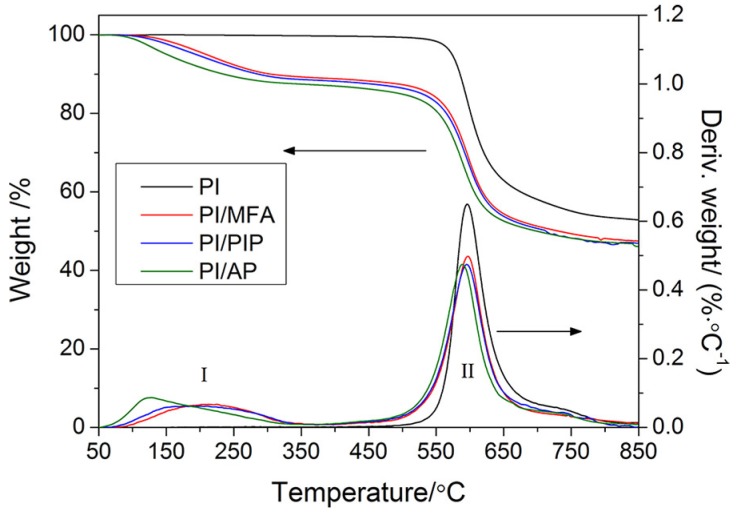
Curves of thermal gravimetric analysis and differential thermal analysis for PI fibers treated with swelling agents (I: the first peak at DTG curve, II: the second peak at DTG curve).

Figure 2 shows the curves of thermal gravimetric analysis (TGA) and differential thermal gravimetric analysis (DTG) for PI fibers treated with swelling agents. The DTG curve of PI fibers shows only one peak at 595.9 °C that can be ascribed to the pyrolysis of PI fibers. The PI fibers exhibited extremely high stability within the temperature range from 50 °C to 350 °C, and only had a weight loss at 350 °C of less than 0.24%. However, two peaks were observed on the DTG curves of PI fibers treated with swelling agents, respectively, being ascribed to the vaporization of swelling agents from the PI fibers and the pyrolysis of PI fibers.

Table 1 summarizes the temperatures of the maximum degradation rate of the PI fibers treated with swelling agents, in which the weight losses at 350 °C of the PI fibers treated with swelling agents are also given for quantifying the amounts of the swelling agents penetrating into the PI fibers. As can be seen in Table 1, the PI fibers treated with swelling agents had a temperature of maximum degradation rate (II) which had changed little. This indicates that the appearance of swelling agents on PI fibers would not significantly impact the thermal properties. The weight loss at 350 °C indicates that swelling agents could penetrate into PI fibers in amounts of 10% or greater pertaining to the amount of PI fibers.

**Figure 3:**
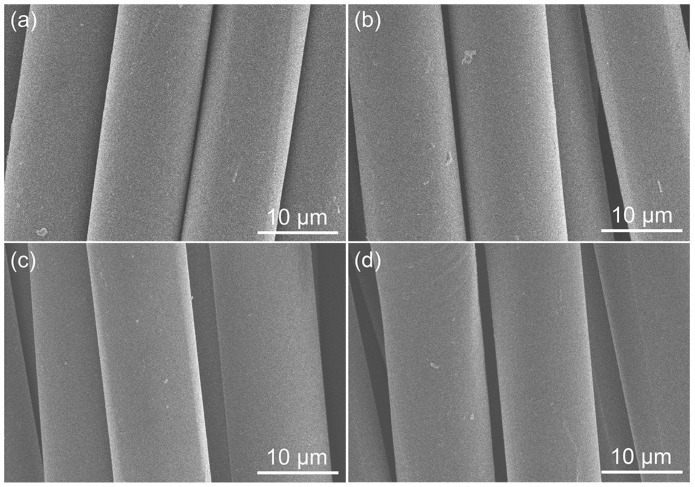
SEM images of PI fibers (**a**) and the PI fibers treated with N-methylformanilide (MFA) (**b**), phenoxyisopropanol (PIP) (**c**), and acetophenone (AP) (**d**).

Figure 3 shows the scanning electron microscope (SEM) images of PI fibers. The treatment with the swelling agents caused no apparent effect on the surface morphology of PI fibers in spite of an amount greater than 10%. This is most likely due to the fact that the swelling agents having smaller molecular sizes than dyes preferentially penetrated into the PI fibers under high temperature conditions, and were embedded into the pore apertures of the PI fibers when cooled down to room temperature. The diameters of PI fibers were slightly increased from 11.7 ± 0.14 μm to 12.06 ± 0.34 μm for treatment with MFA, 12.17 ± 0.10 μm for treatment with PIP, and 12.18 ± 0.25 μm for treatment with AP, respectively. Therefore, the swelling agents did not really swell the PI fibers.

PI fibers were spun into yarns for measurement of the tensile properties as shown in Table 2. It was found that the breaking strength and elongation of the PI yarns decreased more or less when the PI fibers were treated with swelling agents. The decrease of the breaking strength and elongation of PI yarns could be ascribed to the fact that, as the swelling agents penetrated into PI fibers, the interaction forces between the PI macromolecules were replaced by the interaction forces between the polyimide and swelling agent so that the polyimide chains readily slid at a tensile strength.

**Figure 4:**
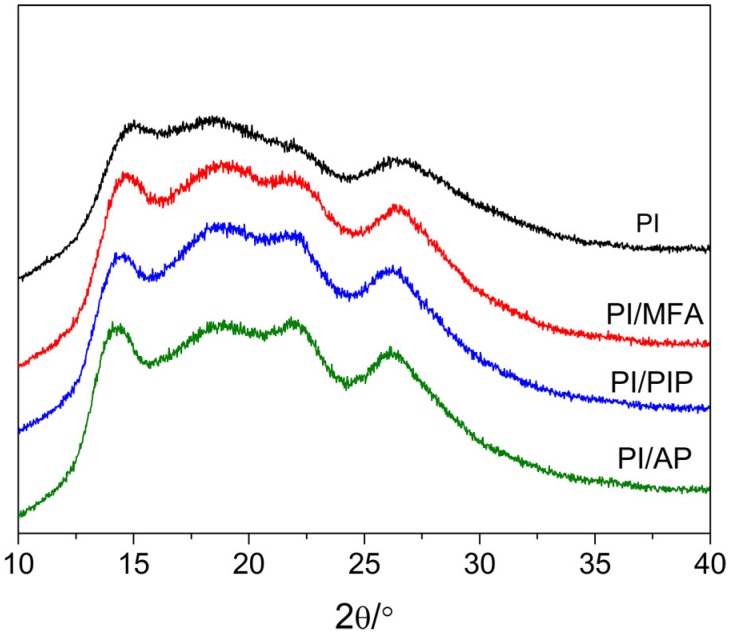
WAXD patterns of PI fibers and PI fibers treated by MFA, PIP and AP.

The results of WAXD analysis are presented in Figure 4. It can be seen that there is a wide diffraction peak accompanied by several peaks on the PI fiber pattern at 14.7°, 22.3°, and 26.5°, corresponding to the crystals of PI fibers. After treated with swelling agents, these three accompanying peaks become sharper, indicating that the crystalliniy of PI fibers increased slightly. This could be ascribed to the solvent-introduced crystallization between fibers and swelling agents. It has been reproted that the interactions between fibers and swelling agents could result in the movements of fiber molecular chains under high-temperature and high-pressure conditions, and crystallinity of the fibers would occur as the temperature increased [21,22]. From Figure 4, however, it can be seen that there is no evident shift of the peaks on the WAXD patterns when PI fibers were treated with swelling agents. This indicates that the crystal form of PI fibers remained almost the same when the PI fibers were treated with the swellling agents.

### 3.2. Effect of Swelling Agents on the Dyeability of PI Fibers

**Figure 5:**
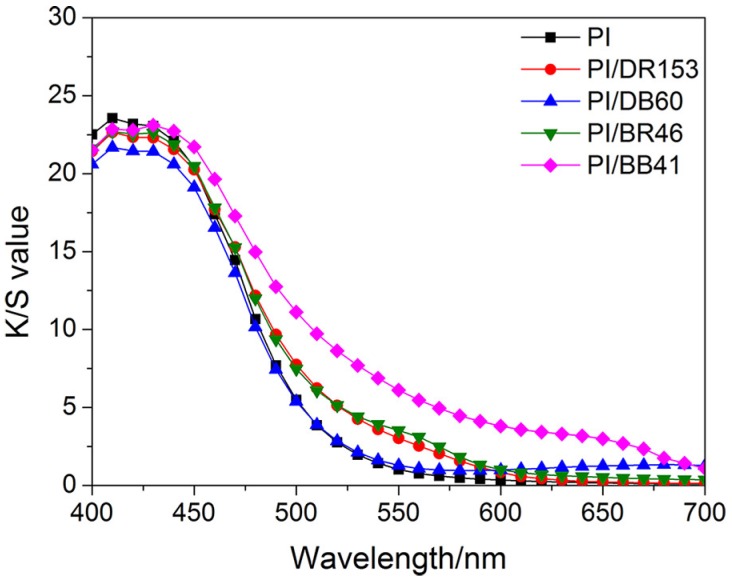
Effect of disperse dyes and basic dyes on the color strengths of PI fibers.

Considering the inherent brilliant golden yellow shade of the PI fibers, red and blue dyes were used for sensitively examining dyeability. Figure 5 shows the color strength of the dyed PI fibers. As can been seen, the PI fibers without dyeing present a strong color strength in the wavelength from 400 nm to 500 nm, which matches their inherent brilliant golden yellow shade. For successful dyeing, the red dyes (DR 153 and BR 46) and blue dyes (DB 60 and BB 41) would need to provide PI fibers with strong color strengths in the wavelength range from 500 nm to 600 nm and the wavelength range from 600 nm to 700 nm, respectively. However, the dyed PI fibers failed to present such strong color strengths as anticipated. This indicates that the PI fibers were hardly dyed with disperse and basic dyes without assistance under high-temperature dyeing conditions (i.e., 130 °C). The poor dyeability of PI fibers is mainly ascribed to the highly compact arrangement of PI macromolecular backbones and the lack of functional groups for dyeing.

**Figure 6:**
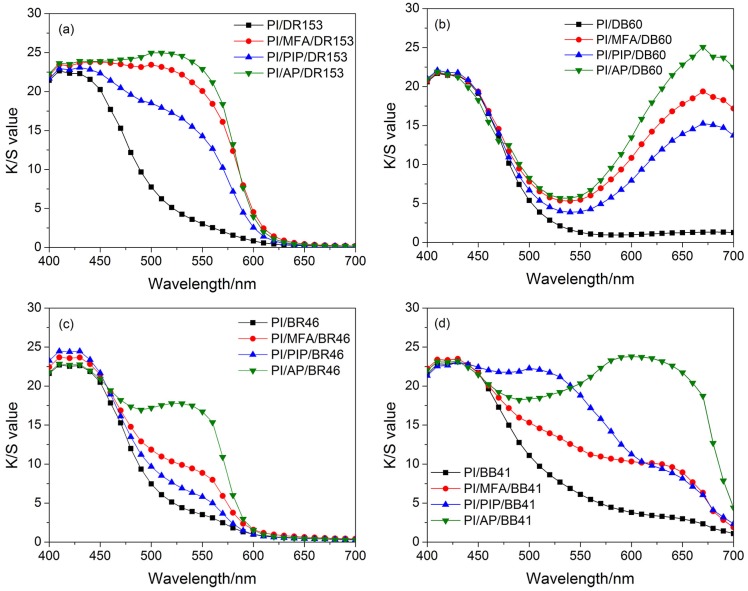
Effect of swelling agents on the color strengths of the PI fibers dyeing with Disperse Red 153 (DR 153) (**a**), Disperse Blue 60 (DB 60) (**b**), Basic Red 46 (BR 46) (**c**), and Basic Blue 41 (BB 41) (**d**).

Figure 6 shows the effect of swelling agents on the color strengths of the dyed PI fibers. It can been seen that the color strengths of the dyed PI fibers were enhanced more or less with the addition of swelling agents. It is thought that the swelling agents enhance the dyeability by interacting with fibers as well as dyes. On the one hand the swelling agents preferentially penetrated into the PI fibers from the dyeing bath to expand the pore apertures of PI fibers through which the dye molecules diffused. On the other hand the swelling agents had a strong solubilization effect on the hydrophobic dyes by which the dye molecules could readily diffuse from the dyeing bath into the PI fibers. Therefore, the dyeability of PI fibers depends on the types of swelling agents as well as the dyes to a great extent. Among the three swelling agents, AP has the highest hydrophobicity, followed by MFA and PIP. According to the similarity-intermiscibility principle, AP could be preferentially adsorbed onto the PI fibers and adequately expand the pore apertures of PI fibers. Additionally, the disperse dyes are more hydrophobic than basic dyes, and more readily adsorbed into the PI fibers than basic dyes with the assistance of the swelling agents. Therefore, it is seen in Figure 6 that, with the addition of swelling agents, the dyeability of the PI fibers was improved more effectively for disperse dyes than basic dyes, and AP was the most effective swelling agent for enhancing the dyeablity of PI fibers, followed by MFA and PIP, with the exception that PIP was used in the dyeing of PI fibers with BB 41. In Figure 6d, it is not to be expected that, when using PIP as the swelling agent, the maximal color strength of the PI fibers dyed with BB 41 would shift to a wavelength of 500 nm. It indicates that PIP most likely interacted with BB 41 so as to result in a change in the chromophore of BB 41.

The PI yarns dyed with the assistance of swelling agents were tested for colorfastness to washing. As shown in Table 3, all the dyed PI yarns exhibited good colorfastness to washing tested by color change. The colorfastness to washing tested by color stain was dependent on dyes to some extent, but did not correlate with swelling agents. This indicates that the PI yarns could be dyed with various color shades with good colorfastness by using swelling agents.

## 4. Conclusions

PI fibers were shown to be difficult to dye with disperse and basic dyes. When treated with swelling agents such as MFA, PIP, and AP, the dyeability of the PI fibers could be significantly enhanced. It was found that swelling agents could penetrate into the PI fibers in amounts greater than 10% (pertaining to the weight of PI fibers). The swelling agent penetrating into the PI fibers did not really swell the PI fibers, but broke the interaction forces between the PI macromolecules. When the swelling agents were used for dyeing of PI fibers, they preferentially penetrated into PI fibers and expanded the pore apertures of PI fibers so that the dye molecules readily diffused into the PI fibers. The swelling agents exhibited different performances in enhancing the dyeablity of PI fibers, and AP was most effective, followed by MFA and PIP. The PI fibers dyed with the assistance of the swelling agents exhibited good colorfastness to washing, tested by color change. The dyed PI fibers’ colorfastness to washing tested by color stain was moderate, which was mainly dependent on the dyes but not the swelling agents. Therefore, using a swelling agent would be an applicable approach for enhancing the dyeability of PI fibers.

## Figures and Tables

**Table 1:** Temperatures of maximum degradation rate of the PI fibers treated with swelling agents and the weight loss at 350 °C.

Sample	Temperature of Maximum Degradation Rate (°C)		Weight Loss at 350 °C (%)
	I	II	
Polyimide (PI)	None	595.9	0.24
PI/N-methylformanilide (MFA)	213.1	596.9	10.72
PI/Phenoxyisopropanol (PIP)	209.6	595.2	11.36
PI/Acetophenone (AP)	128.3	588.8	12.55

**Table 2:** Tensile properties of PI yarns treated with swelling agents.

Yarn	Breaking Strength (cN/tex)	Breaking Elongation (%)
PI	25.32	9.78
PI/MFA	20.53	8.04
PI/PIP	20.43	7.84
PI/AP	21.16	9.41

**Table 3:** Colorfastness to washing of PI fabrics dyed with the assistance of swelling agents.

Dye	Swelling Agents	Color Change	Color Stain					
			Wool	Acrylic	Polyester	Polyamide	Cotton	Acetate
DR 153	MFA	4–5	3	4–5	3–4	3	4	3
	PIP	4–5	3	3–4	2–3	2	3	2–3
	AP	4–5	3–4	4	3	2–3	3–4	3
DB 60	MFA	5	5	5	5	5	5	5
	PIP	5	5	5	5	5	5	5
	AP	5	5	5	5	4	5	5
BR 46	MFA	5	5	5	5	5	3–4	5
	PIP	5	4–5	5	5	4–5	3	4–5
	AP	4–5	4	4–5	4	4–5	3	4–5
BB 41	MFA	5	5	5	5	4	5	4–5
	PIP	4–5	4	5	4	3	3	3
	AP	5	4–5	5	4–5	3–4	3	3–4
